# *Phyllodistomum kupermani* n. sp. from the European perch, *Perca fluviatilis* L. (Perciformes: Percidae), and redescription of *Phyllodistomum macrocotyle* (Lühe, 1909) with notes on the species diversity and host specificity in the European *Phyllodistomum* spp. (Trematoda: Gorgoderidae)

**DOI:** 10.1186/s13071-020-04434-2

**Published:** 2020-11-10

**Authors:** Romualda Petkevičiūtė, Alexander E. Zhokhov, Virmantas Stunžėnas, Larisa G. Poddubnaya, Gražina Stanevičiūtė

**Affiliations:** 1 Institute of Ecology of Nature Research Centre, Akademijos str. 2, LT-08412 Vilnius, Lithuania; 2 Papanin Institute for Biology of Inland Waters, Russian Academy of Sciences, Borok, Russia

**Keywords:** *Phyllodistomum kupermani* n. sp., *Phyllodistomum macrocotyle*, European perch, ITS2 rDNA, *28S*, Host specificity, SEM, Morphological variation

## Abstract

**Background:**

European species of the large genus *Phyllodistomum* Braun, 1899 had historically been erected based solely on morphological characters. Unfortunately, many of them are still poorly known and inadequately described. Molecular approaches are critical to delineate species which were impossible to differentiate based on morphology alone.

**Methods:**

New samples of adult *Phyllodistomum* spp. were collected from the urinary bladder and/or ureters of European freshwater fishes and fixed to conduct a light and scanning electron microscopy study, and to obtain sequences of nuclear (ITS2 spacer and *28S* rRNA gene), to be analysed in the context of a molecular phylogeny.

**Results:**

Based on new findings, a new species of *Phyllodistomum* from the urinary bladder of the European perch, *Perca fluviatilis*, in Volga River basin, Russia, is described. Additionally, new data on the morphology and tegumental surface topography of *P. macrocotyle* (Lühe, 1909) Odhner, 1911 from ureters of the common rudd, *Scardinius erythrophthalmus*, is presented. The host range of *P. folium*, confirmed by DNA analysis, is extended to other cyprinid fish species.

**Conclusions:**

The present study has again shown that species of the genus *Phyllodistomum* are in dire need of revision based on both molecular analysis and detailed morphological redescriptions of the forms attributed to the genus. Morphologically, *P. kupermani* n. sp. most closely resembles *P. pseudofolium*, a highly host-specific parasite of *Gymnocephalus cernuus* (L.), but molecular phylogenetic analyses based on ITS2 and *28S* rDNA sequences showed that these species are distantly related. *Phyllodistomum kupermani* n. sp. was found to be phylogenetically most closely related to the type-species of *Phyllodistomum*, *P. folium.* Phylogenetic analyses revealed that *Phyllodistomum kupermani* n. sp. and *P. folium* formed a clade with other freshwater species for which cystocercous cercariae develop in bivalves of the family Sphaeriidae. The micromorphology and tegumental surface topography of *P. macrocotyle* revealed in the present study provide a valuable taxonomic criterion for congeneric species differentiation.
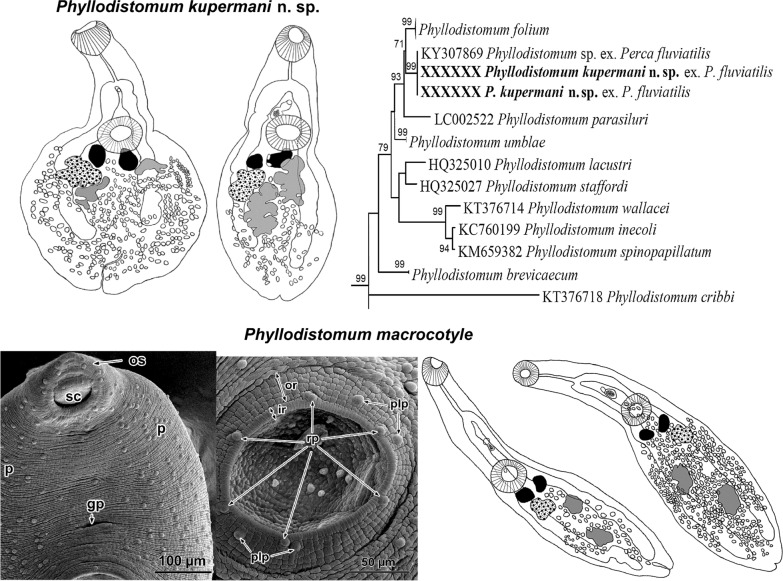

## Background

*Phyllodistomum* Braun, 1899 is one of the most speciose digenean genera, comprised of species parasitizing in the urinary bladder and/or ureters of freshwater and marine fish and, more rarely, amphibians throughout the world [1–6], and new species descriptions continue to be published on a regular basis [3, 4, 7, 8]. Species of *Phyllodistomum* infecting fishes of Europe have been studied for more than two centuries, starting with the description of *Phyllodistomum umblae* (Fabricius, 1780) (as *Fasciola umblae*) from the Arctic char, *Salvelinus alpinus* (L.) in Norway. Despite the relatively common occurrence of *Phyllodistomum* spp. in European freshwater fishes, the species composition of the genus is under scrutiny and remains controversial. Our recent studies [5, 6, 9] have challenged previous data on species diversity and life-cycles in this presumably well-known group of trematodes. However, there are still serious gaps in our knowledge of the genus *Phyllodistomum* and a number of unanswered questions concerning the validity and specificity of the nominal species and the identity of unidentified genetic lineages are still awaiting clarification.

Previous molecular phylogenetic analysis revealed that specimens of *Phyllodistomum* sp. (preliminary identified as *P. pseudofolium* Nybelin (1926)) obtained from the urinary bladder of the European perch, *Perca fluviatilis* L., from Volga River basin, Russia, represent distinct genetic lineage (presumably an undescribed species), resolving between *P*. *folium* (Olfers, 1816) and *P. umblae* clades in the ITS2 and *28S* phylograms [6]. To validate the independent taxonomic status of this genetic linage, a comparative morphological study of *Phyllodistomum* species from *P. fluviatilis* was required. New samples of *Phyllodistomum* spp. were also obtained from the urinary system of three species of cyprinids, the ide, *Leuciscus idus* (L.), the common rudd, *Scardinius erythrophthalmus* (L.) and the common roach, *Rutilus rutilus* (L.). Morphological characteristics along with sequence data allowed us to identify these samples to species. The present paper provides description of a new species of *Phyllodistomum* and a detailed redescription of the adult stage of *P. macrocotyle* (Lühe, 1909) Odhner, 1911 based on both light microscopy (LM) and scanning electron microscopy (SEM). *Phyllodistomum macrocotyle* has a complex taxonomic history and this name has often not been used properly. The microcercous cercariae of *P. macrocotyle* develop in sporocysts localised the gills of the intermediate host *Dreissena polymorpha*. In the past, *Phyllodistomum* trematodes found in *D. polymorpha* were also referred to as *P. folium sensu* Sinitsin, 1905 or *P. dogieli* Pigulevsky, 1953 but recent molecular evidence has shown that *P. macrocotyle* is the only valid *Phyllodistomum* species thus far documented from *D. polymorpha* [5]. It is notable that Pigulevsky [10], without convincing reasons, named this species *P. dogieli*; hence, both *P. dogieli* and *P. folium sensu* Sinitsin, 1905 should be regarded as synonymous with *P. macrocotyle*. To avoid further confusion, morphological redescription of adults is required in combination with molecular data. It should be noted that the study of Peribáñez et al. [11] used comparative analysis of ITS1-*5.8S*-ITS2 sequence data in order to link digeneans (named as *P. folium*) from the urinary system of three cyprinid species, *S. erythrophthalmus*, *Cyprinus carpio* and *R. rutilus*, and the sporocysts found in zebra mussels, *D. polymorpha*, in the Ebro River, Spain. However, adult digeneans were not observed by microscopic examination and no morphological characteristics of these specimens are available from this study.

Recent progress shows again that species delimitation will require the use of molecular markers in combination with morphological description to discriminate species and verify validity of problematic nominal species. Continuing with our efforts to survey *Phyllodistomum* species diversity in European freshwater fishes, we describe the new species with sequences identical to *Phyllodistomum* sp. previously reported by Stunžėnas et al. [6] from Russia. Also, we provide a detailed morphological description of *P. macrocotyle*, including microphotographs of the body surface though SEM, which is for the first time linked to ITS2 and *28S* rDNA sequences.

## Methods

Specimens of *Phyllodistomum* were recovered from the ureters and urinary bladder of the European perch, *Perca fluviatilis*, the ide, *Leuciscus idus*, the common rudd, *Scardinius erythrophthalmus*, the common roach, *Rutilus rutilus* from Volga River basin, Russia (Table 1).

**Table 1:** Species subjected to molecular phylogenetic analysis with information of their host, locality and GenBank accession numbers

Species	Host	Locality	GenBank ID [References]	
			*28S*	*5.8S*-ITS2-*28S*
*Cercaria duplicata*	*Anodonta anatina*	Lake Saravesi, Finland	KJ729516 [5]	KJ740490 [5]
*C. duplicata*	*A. anatina*	Kaunas water Reservoir, Lithuania	KJ729515 [5]	KJ740492 [5]
*C. duplicata*	*A. anatina*	River Sluch, Ukraine	KJ729517 [5]	KJ740489 [5]
*Phyllodistomum folium*	*Esox lucius*	River Ild, Russia	KJ729542 [5]	KJ740500 [5]
*P. folium*	*Rutilus rutilus*	Rybinsk water Reservoir on the Volga River, Russia	KJ729536 [5]	KJ740501 [5]
*P. folium*	*Gymnocephalus cernuus*	River Chesnava near Rybinsk water Reservoir on the Volga River, Russia	KX957728 [6]	KY307883 [6]
*P*. *folium*	*Cottus gobio*	River Nėris, Lithuania	KJ729550 [5]	KJ740507 [5]
*P. folium*	*Gasterosteus aculeatus*	River Vilnelė, Lithuania	AY277707 [42]	AY277705 [42]
*P. folium*	*Scardinius erythrophthalmus*	Rybinsk water reservoir on the Volga river, Russia	**MT872646**	
*P. folium*	*Rutilus rutilus*	River Sunoga, Russia	**MT872645**	
*P. folium*	*Sphaerium corneum*	River Shumorovka, Russia	KJ729546 [5]	KJ740498 [5]
*P. folium*	*S. corneum*	River Hegga, Norway	KJ729551 [5]	KJ740495 [5]
*P. folium*	*Pisidium supinum*	River Ūla, Lithuania	KJ729544 [5]	KJ740496 [5]
*P. folium*	*Pisidium amnicum*	River Ild, Russia	KJ729535 [5]	KJ740497 [5]
*Phyllodistomum umblae*	*Pisidium hibernicum*	Lake Nordersjoen, Norway	KP284109, KP284110 [9]	
*P. umblae*	*Coregonus albula*	Lake Syamozero, Karelia, Russia	KJ729528 [5]	KJ740508 [5]
*Phyllodistomum angulatum*	*Sander lucioperca*	River Chesnava, Russia		KJ740511, KJ740512 [5]
*P. angulatum*	*S. lucioperca*	Rybinsk water reservoir on the Volga river, Russia	KX957734 [5]	
*Phyllodistomum pseudofolium*	*G. cernuus*	River Chesnava near Rybinsk water reservoir on the Volga river, Russia	KX957732 [6]	KY307875 [6]
*P. pseudofolium*	*P. amnicum*	River Chesnava, Russia		KJ740513 [5]
*P. pseudofolium* (syn. *Phyllodistomum* sp. of Ginetzinskaya (1959))	*P. amnicum*	Lithuania	AY281126 [42]	
*Phyllodistomum* sp.	*Perca fluviatilis*	Rybinsk water reservoir on the Volga river, Russia	KY307869 [6]	KY307886 [6]
*Phyllodistomum kupermani* n. sp.	*P. fluviatilis*	Rybinsk water reservoir on the Volga river, Russia	**MT875008, MT875009**	**MT875012, MT875013**
*Phyllodistomum macrocotyle*	*Dreissena polymorpha*	Lake Vilkokšnis, Lithuania;	KJ729518 [5]	KJ740518 [5]
	*D. polymorpha*	Lake Kretuonas, Lithuania		KJ740514 [43]
*P. macrocotyle* (syn. *P. folium sensu* Sinitsin, 1905)	*D. polymorpha*	Lake Lepelskoe, Belarus	AY288828 [43]	AY288831 [43]
*P. macrocotyle* (syn. *P. folium sensu* Sinitsin, 1905)	*D. polymorpha*	Lake Lukomskoe, Belarus	AY281127 [43]	AF533015 [43]
*P. macrocotyle*	*Scardinius erythrophthalmus*	Rybinsk water reservoir on the Volga river, Russia	**MT872664**	**MT875011**
*P. macrocotyle*	*Leuciscus idus*	Rybinsk water reservoir on the Volga river, Russia	**MT872663**	**MT875010**
*Phyllodistomum magnificum*	*Tandanus tandanus*	Moggill Creek, Queensland, Australia	KF013189 [34]	KF013153, KF013156 [34]
*Phyllodistomum inecoli*	*Heterandria bimaculata*	Agua Bendita, Xico, Veracruz, Mexico	KC760199 [41]	
*Phyllodistomum lacustri*	*Noturus flavus*	Canada	HQ325010 [44]	
*Phyllodistomum parasiluri*	*Silurus asotus*	Japan	LC002522 [35]	
*Phyllodistomum spinopapillatum*	*Profundulus balsanus*	Rio PuebloViejo, Mexico	KM659382 [8]	
*Phyllodistomum staffordi*	*Ameiurus melas*	Canada	HQ325027 [44]	
*Phyllodistomum wallacei*	*Xenotaenia resolanae*	Mexico	KT376714 [4]	
*Phyllodistomum centropomi*	*Centropomus parallelus*	Tlacotalpan, Veracruz, Mexico	KM659384 [8]	
*Phyllodistomum cribbi*			KT376718 [4]	
*Phyllodistomum kanae*	*Hynobius retardatus*	Pippu, Hokkaido, Japan	AB979868 [3]	
*Phyllodistomum brevicecum*	*Umbra limi*	Brokenhead, Manitoba, Canada	KC760207 [41]	
*Phyllodistomum brevicaecum*	*U. limi*	Canada	HQ325008 [44]	
*Phyllodistomum* cf*. symmetrorchis*	*Clarias gariepinus*	Lake Victoria, Kenya	KF013171 [34]	KF013162 [34]
*Phyllodistomum vaili*	*Mulloidichthys flavolineatus*	Lizard Island, Queensland, Australia	KF013173 [34]	KF013155 [34]
*Phyllodistomum hoggettae*	*Plectropomus leopardus*	Lizard Island, Queensland, Australia	KF013191 [34]	KF013148 [34]
*Phyllodistomum hyporhamphi*	*Hyporhamphus australis*	Australia	KF013190 [34]	KF013150 [34]
*Phyllodistomum* sp.	*Cephalopholis boenak*	Australia	KF013175 [34]	
*Phyllodistomum* sp.	*Epibulus insidiator*	French Polynesia	KF013179 [34]	
*Phyllodistomum pacificum*	*Pantolabus radiatus*	Moreton Bay, Queensland, Australia	MG845599 [45]	MG845601 [45]
*Pseudophyllodistomum johnstoni*	*Macrobrachium australiense*	Warrill Creek, Queensland, Australia	KF013177 [34]	KF013166 [34]
*Gorgodera cygnoides*	*Rana ridibunda*	Switzerland	AF151938 [46]	
*G. cygnoides*	*R. ridibunda*	Kokaljane, near Sofia, Bulgaria	AY222264 [16]	
*Gorgodera amplicava*	*Rana catesbeiana*	Nebraska, USA		FJ445743 [47]
*Gorgoderina attenuata*	*Rana clamitans*	Nebraska, USA		FJ445741 [47]
*Gorgoderina simplex*	*R. clamitans*	Nebraska, USA		FJ445742 [47]
*Gorgoderina lufengensis*	*Nanorana yunnanensis*	China	MH277507, Ding J., unpublished	MH257738, Ding J., unpublished
*Xystretrum solidum*	*Sphoeroides testudineus*	Conch Key, Florida, USA	KF013188 [34]	KF013149 [34]
*Xystretrum caballeroi*	*Balistes polylepis*	Mexico	HQ325030 [44]	
*Xystretrum* sp.	*Sufflamen fraenatum*	Ningaloo, Western Australia, Australia		KF013160 [34]
Outgroup				
*Nagmia floridensis*	*Rhinoptera bonasus*	Gulf of Mexico, East Ship Island, Mississippi, USA	AY222262 [18]	
*Cercariaeum crassum*	*P. amnicum*	Finland: Siilaisenpuro River	JF261141. [48]	
*Bucephalus polymorphus*	*D. polymorpha*	Belarus	AY289248 [43]	AY289239 [43]
*B. polymorphus*	*P. fluviatilis*	Curonian Lagoon, Lithuania		JQ346725 [13]
*Maritrema arenaria*	*Semibalanus balanoides*	Belfast Lough, Northern Ireland		HM584171 [49]

*Note*: Sequences generated in the present study are indicated in bold

Adult trematodes were isolated from the urinary system of naturally infected fish, placed in saline solution (0.65%) and identified *in vivo*. Specimens selected for DNA extraction were washed in saline and preserved in 96% ethanol. Voucher specimens from the same collecting event used for morphological examination were washed in saline, fixed without pressure in hot 10% formalin. Morphological samples were stained with alum carmine, dehydrated in a graded ethanol series, cleared in clove oil and mounted as permanent slides using Canada balsam. Voucher specimens are deposited in the helminthological collection of I. D. Papanin Institute of Biology of Inland Waters, Russia. Extracted total DNA is deposited in the P. B. Šivickis Laboratory of Parasitology of Nature Research Centre, Vilnius, Lithuania.

For SEM, 10 live specimens of *P. macrocotyle* from *S. erythrophthalmus* were fixed in 3% glutaraldehyde in 0.1 m sodium cacodylate buffer (pH 7.2) for 20 days at 5 °C and then dehydrated in a graded ethanol series, with a final change to absolute acetone. They were then critical-point dried with liquid CO_2,_ mounted on stubs, sputter-coated with gold-palladium and examined using a JEOL JMS 6510LV scanning electron microscope operating at 30 kV. Genomic DNA was extracted from ethanol-fixed specimens according to the protocol of Stunžėnas et al. [12] with slight modifications [13]. The internal transcribed spacer 2 region (ITS2) was amplified using the forward primer 3S (5′-CGG TGG ATC ACT CGG CTC GTG-3′) [14] and the reverse primer ITS2.2 (5′-CCT GGT TAG TTT CTT TTC CTC CGC-3′) [15]. A new primer pair, GoJe-F and GoJe-R, was designed for species of the Gorgoderidae. Part of the internal transcribed spacer 1 (ITS1), the complete *5.8S* rDNA and ITS2, also a small section at the 5’-end of the *28S* gene were amplified using forward primer GoJe-F (5′-CTT GCA ATT GTT CCC CGT GA-3′) and the reverse primer GoJe-R (5′-CTG TTC ACT CGC CGT TAC TG-3′). A fragment at the 5′-end of the *28S* rRNA gene was amplified using forward primers Digl2 (5′-AAG CAT ATC ACT AAG CGG-3′) or ZX-1 (5′-ACC CGC TGA ATT TAA GCA TAT-3′) [16] and reverse primers L0 (5′-GCT ATC CTG AG (AG) GAA ACT TCG-3′) [17] or 1500R (5′-GCT ATC CTG AGG GAA ACT TCG-3′) [18]. The new primer pair, GoJe-F and GoJe-R, were utilised under the following conditions: initial denaturation for 3 min at 96 °C, 38 cycles of 28 s at 95 °C, 38 s at 52 °C, 38 s at 72 °C, and a final extention step for 8 min at 72 °C. The amplification protocols for other primers are as described in our previous study [13]. PCR products were sequenced in both directions at BaseClear B.V. (Leiden, the Netherlands) using PCR primers. Contiguous sequences were assembled using Sequencher 4.7 software (Gene Codes Corporation). The newly generated sequences of *P. macrocotyle*, *P. folium* and the new *Phyllodistomum* species, were deposited on GenBank (see accession numbers in Table 1).

Additional rDNA sequences of gorgoderid species and outgroup taxa (Table 1) were downloaded from GenBank and included in pairwise sequence comparisons and phylogenetic analyses. For the phylogenetic analyses, both the ITS2 and *28S* datasets were aligned using ClustalW [19] with an open gap penalty of 15 and gap extension penalty of 6.66. The best-fit model for phylogenetic analysis was estimated using jModeltest v. 0.1.1 software [20]. Ambiguously aligned positions were excluded from phylogenetic analysis. Maximum Likelihood (ML) phylogenetic trees were obtained and analyzed using MEGA v6 [21]. Branch support was estimated by bootstrap analyses with 1000 pseudoreplicates. The ML trees were obtained using the general time reversible model with a gamma distribution rate and a proportion of invariant sites (GTR + G + I) for both the ITS2 and the *28S* gene datasets. Gamma shape and the number of invariant sites were estimated from the data. Parsimony analysis based on subtree pruning and regrafting (SPR) was used with default parsimony settings. If two or more sequences belong to one species, they were collapsed into one branch, except those of the new species and *P. macrocotyle.* Estimates of mean evolutionary divergence over sequence pairs within and between groups were calculated using the MEGA v6 programme.

## Results

### General results

*Perca fluviatilis* was rarely infected with *Phyllodistomum* in the water bodies studied; only nine gravid specimens were recovered form 25 fish examined in 2018. The molecular studies demonstrated that these specimens belong to a new species. Of the 30 individuals of *S. erythrophthalmus* studied, 30% were infected with 1–7 phyllodistomes per fish. Trematodes recovered from the ureters of the host fishes have been shown using molecular methods to be *P. macrocotyle*, while specimens inhabiting the urinary bladder were identified as *P. folium*. Trematodes from the ureters of *L. idus* and *R. rutilus*, were identified as *P. macrocotyle* and *P. folium*, respectively. It is noteworthy that all specimens inhabiting ureters of the studied cyprinid fishes morphologically closely resembled *P. elongatum* Nybelin, 1926.

### Phylogenetic analysis

Sequence data for two regions of rDNA, the *5.8S*-ITS2-*28S* and 5’-end of the *28S* gene were used for species-level comparison among samples of *Phyllodistomum* spp. and to determine phylogenetic affinities of the different morphotypes from different host species.

**Fig. 1:**
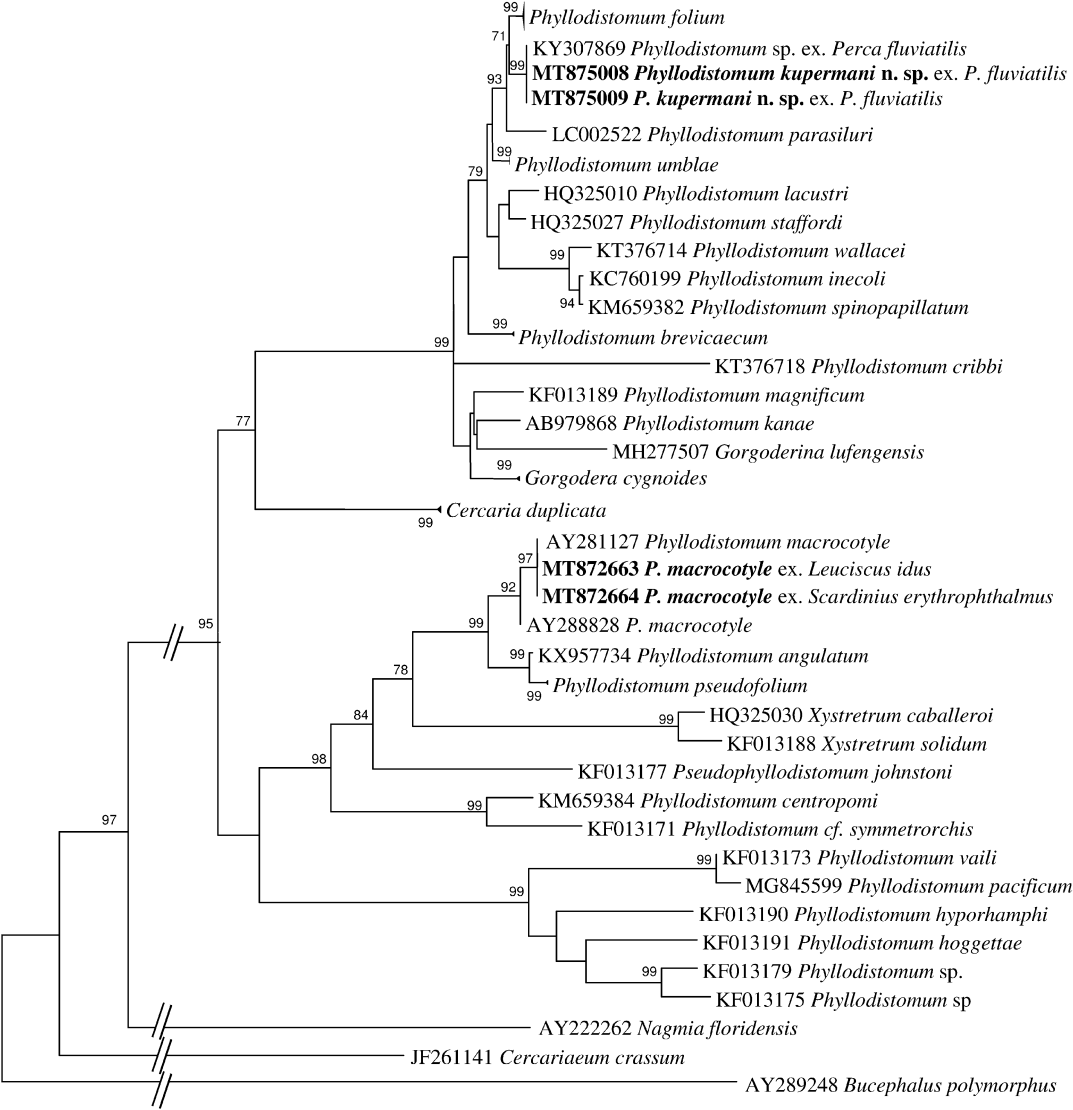
Phylogenetic tree based on Maximum Likelihood analysis of partial sequences of the 28S nuclear rDNA gene. Bootstrap support values lower than 70% are not shown. The species sequenced in this study are indicated in bold. GenBank accession numbers of the collapsed clades are provided in Table 1

**Fig. 2:**
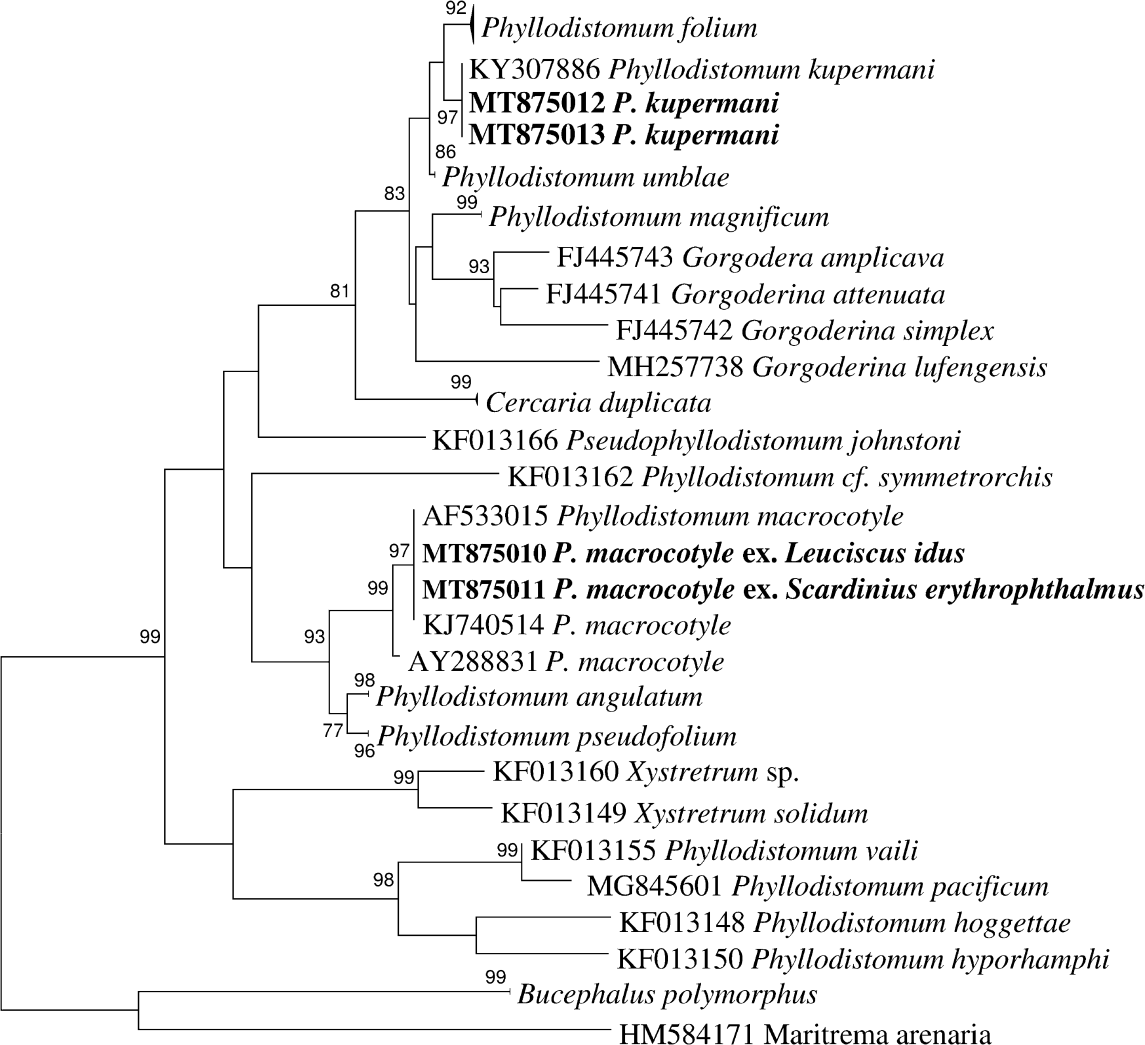
Phylogenetic tree based on Maximum Likelihood analysis of the ITS2 nuclear rDNA region. Bootstrap support values lower than 70% are not shown. The species sequenced in this study are indicated in bold. GenBank accession numbers of the collapsed clades are provided in Table 1

Alignment of the ITS2 rDNA and *28S* rDNA regions for gorgoderid species yielded 495 and 836 characters for phylogenetic analysis, respectively. New sequences of *P. folium* were identical to those previously reported from other fish hosts. New rDNA sequences for adult *P. macrocotyle* and sequences of larval stages from *Dreissena polymorpha* (Pallas, 1771) from a previous study [5] formed a strongly supported subclade nested in a well-supported monophyletic clade including *P. pseudofolium* and *P. angulatum* Linstow, 1907. The sequences of the new species were identical to *Phyllodistomum* sp. ex *P. fluviatilis* from our previous study [6] and formed a sister clade with sequences of *P. folium* in both *28S* and ITS2 phylograms (Figs. 1, 2). Notably, in the *28S*-based phylogeny, *P. parasiluri* Yamaguti, 1934 was sister to the clade constituted by *P. folium* and *P. kupermani* n. sp. The genetic distance values from the *28S* and ITS2 datasets of the new species, when compared with the most closely related species *P. folium*, were 1.1‒1.3% and 1.2‒1.4%, respectively. The new species differed from *P. umblae* by 1.8% for *28S* and 1.2% for ITS2. With respect to the most morphologically similar species, *P. pseudofolium*, the divergence was much greater: 13% (*28S*) and 11% (ITS2). New sequences of adult *P. macrocotyle* from *S. erythrophthalmus* and *L. idus* appeared to be identical to the sequences of larval *P. macrocotyle* from its first intermediate host *D. polymorpha.*

### Morphological description

**Family Gorgoderidae Looss, 1899**

**Genus** ***Phyllodistomum*** **Braun, 1899**

***Phyllodistomum kupermani*** **n. sp.**

***Type-host***: *Perca fluviatilis* L. (Perciformes: Percidae).

***Type-locality***: River Ilʼdʼ (58°00ʹ48ʺN, 38°09ʹ54ʺE), Yaroslavl Province, Russia.

***Other locality***: River Latka (58°06ʹ17ʺN, 38°13ʹ53ʺE), Yaroslavl Province, Russia.

***Type-material***: The type-specimens are deposited in the Parasite Collection of the Institute for Biology of Inland Waters RAS, Russia: holotype No. 1/13 (1), paratypes: No. 1/13 (2–6).

***Site in host***: Urinary bladder.

***Representative DNA sequences***: *28S* rDNA (MT875008-MT875009); ITS2 rDNA (MT875012-MT875013).

***ZooBank registration***: To comply with the regulations set out in Article 8.5 of the amended 2012 version of the *International Code of Zoological Nomenclature* (ICZN) [22], details of the new species have been submitted to ZooBank. The Life Science Identifier (LSID) of the article is urn:lsid:zoobank.org:pub:607C4D81-BB93-4BAC-9C1A-44F63F1A46A5. The LSID for the new name *Phyllodistomum kupermani* is urn:lsid:zoobank.org:act:1D8C1A16-ADB1-47FD-9678-369906F9C6C6.

***Etymology***: The new species is named after Professor Boris I. Kuperman (1933–2002), Institute for the Biology of Inland Waters (IBIW) of the Russian Academy of Sciences and San Diego State University, California, in recognition of his great contribution to our knowledge on biology, evolution, ecology, functional morphology and biochemistry of fish parasites.

### Description

**Fig. 3:**
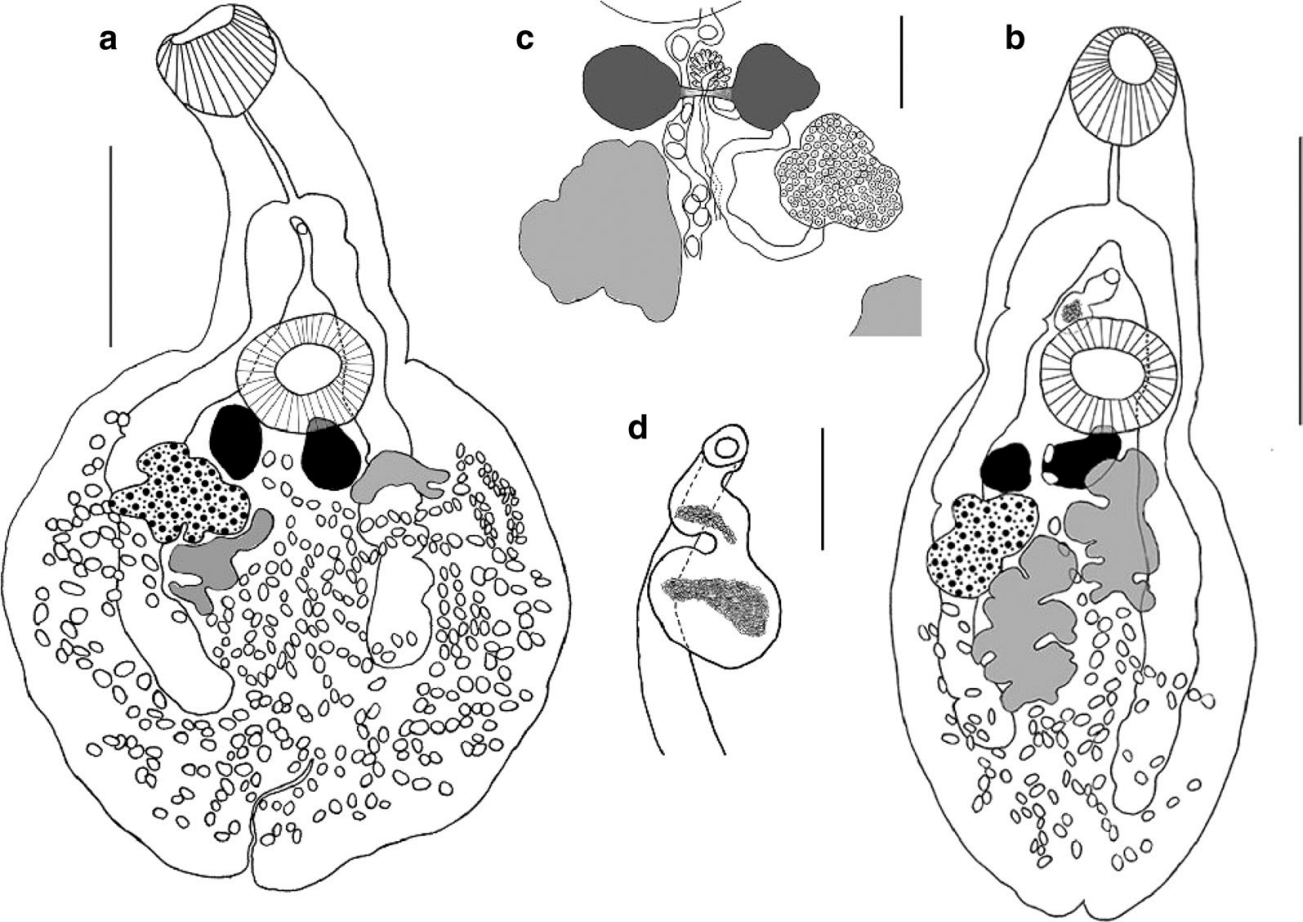
*Phyllodistomum kupermani* n. sp. ex *Perca fluviatilis*. **a** Holotype, whole-mount ventral view. **b** Paratype, whole-mount ventral view. **c** Paratype, detail of male and female reproductive complex. **d** Paratype, terminal genitalia. Scale-bars: **a**, **b** 500 μm; **c** 100 μm; **d** 50 μm

[Based on 10 gravid specimens; Fig. 3] Body pear-shaped in young specimens or scoop-shaped in old specimens, covered with minute spines, 1332–1872 (1515 ± 198) long, maximum width 432–1188 (750 ± 261) at level of middle region of hindbody. No marginal folds present. Body length to width ratio 1.5–3.1 (2.2). Forebody short, 396–666 (495 ± 87) long, representing 28–38% (33%) of total body length. Hindbody leaf-shaped in young specimens and discoid in older ones, 882–1296 (1056 ± 161) long. Oral sucker globular, subterminal or terminal, 144–210 (176 ± 22) long, 132–210 (173 ± 25) wide. Ventral sucker round, larger than oral sucker, 192–300 (236 ± 35) long, 180–300 (235 ± 42) wide. Oral sucker length to width ratio 1:0.69–0.81 (1:0.73). Ventral sucker length to width ratio 1:0.67–0.89 (1:0.74). Oral sucker to ventral sucker distance 198–510 (344 ± 83). Pharynx absent. Oesophagus straight, 102–210 (168 ± 36) long. Intestinal bifurcation 306–426 (357 ± 49) from anterior extremity. Caeca wide, terminating conspicuously far from posterior extremity, of unequal lengths, 255–441 (324 ± 69) from posterior extremity.

Gonads and vitelline masses closely packed. Testes 2, moderately or deeply lobed, oblique. Anterior testis 132–252 (208 ± 38) long, 99–300 (159 ± 63) wide; posterior testis 180–360 (272 ± 71) long, 117–300 (164 ± 61) wide. Anterior testis is situated very closely to vitellarium, posterior testis is situated very closely to ovary, distance between posterior testis and ovary 0–60 (22 ± 20). Seminal vesicle saccular, relatively short, convoluted, 44–110 (85 ± 25) long, 44–77 (58 ± 13) wide. Pars prostatica not observed. Genital pore median, at level of caecal bifurcation, 55–270 (121 ± 73) from anterior margin of ventral sucker.

Ovary irregular, moderately lobed, dextral in 7 specimens, sinistral in 3. Mehlisʼ gland dorsal, posterior to ventral sucker, between vitelline masses. Vitelline masses 2, compact, entire or slightly lobed, round or oval, between ovary and ventral sucker; right vitelline mass 57–156 (100 ± 31) long, 55–150 (93 ± 33) wide; left vitelline mass 84–156 (104 ± 23) long, 48–132 (81 ± 24) wide. Uterus extensively coiled, occupying entire hindbody, inter- and extracaecal. Eggs oval, 29–35 × 20–25 (33 × 22). Excretory vesicle I-shaped, extending to level of caeca end. Excretory pore terminal.

### Remarks

The present material most closely resembles *P. pseudofolium*, a species described from ureters of the Eurasian ruffe, *Gymnocephalus cernuus*, and strictly specific to its definitive host [6, 10]. However, the new species differs from *P. pseudofolium* in its hindbody shape (has no body fold or other demarcation), and in having a narrower oral sucker and a larger ventral sucker, deeply lobed testes and a short distance between the posterior testis and ovary.

Our specimens can be distinguished from the other apparently valid European *Phyllodistomum* spp. (*P. folium*, *P. angulatum*, *P. umblae*, *P. macrocotyle* and *P. elongatum*). *Phyllodistomum kupermani* n. sp. differs from *P. folium* in the oval shape of the vitelline masses and the closely packed gonads and vitelline masses. The present material differs from *P. angulatum* by having a smaller body size (*P. angulatum* is almost 2 times larger), a short forebody and the absence of mid-ventral lateral muscular flaps (a typical diagnostic character for *P. angulatum* when alive). The new species differs from *P. umblae* in the body shape and size, caeca length and size of eggs. *Phyllodistomum umblae* has an elongated shape with the longer foliate hindbody, long caeca, which terminate close to the posterior extremity. *Phyllodistomum umblae* is a large worm (2990 *vs* 1515 μm) and has larger eggs (37 × 27 *vs* 33 × 22 μm). In addition, *P. umblae* is a parasite of salmonid fishes. The new species differs from *P. macrocotyle* in its body shape and the arrangement of the genital pore. *Phyllodistomum macrocotyle* has an elongated form and can be distinguished from *P. kupermani* n. sp. by its lanceolate hindbody, longer cylindrical forebody representing 36% of total body length, genital pore midway between caecal bifurcation and ventral sucker margin. *Phyllodistomum kupermani* n. sp. can be clearly distinguished from *P. elongatum* by its body shape, the arrangement of the genital pore and the shape of vitelline masses. *Phyllodistomum elongatum* has a more elongated body shape with lanceolate hindbody, a genital pore situated pore more posteriorly (near to ventral sucker margin) and lobed vitelline masses. In addition, *P. elongatum* and *P. macrocotyle* are located only in the ureters of the cyprinid fish.

***Phyllodistomum macrocotyle*** **(Lühe, 1909) Odhner, 1911**

Syns *Catoptroides macrocotyle* Lühe, 1909; *Phyllodistomum folium sensu* Sinitsin, 1905 *nec* Olfers, 1817; *Phyllodistomum dogieli* Pigulevsky, 1953.

***Type-host***: *Scardinius erythrophthalmus* (L.) (Cypriniformes: Cyprinidae).

***Other host***: *Leuciscus idus* (L.) (Cypriniformes: Cyprinidae) (present study).

***Type-locality***: Rybinsk Reservoir (58°02ʹ28ʺN, 38°15ʹ18ʺ E), Yaroslavl Province, Russia.

***Voucher material***: Eleven voucher specimens ex *S. erythrophthalmus* on 8 slides [No. 1/12(1–8)] were deposited in the Parasite Collection of the Institute for Biology of Inland Waters RAS, Russia.

***Site in host***: Ureters.

***Representative DNA sequences***: *28S* rDNA (MT872663-MT872664); ITS2 rDNA (MT875010-MT875011) (see also Table 1).

### Redescription

**Fig. 4:**
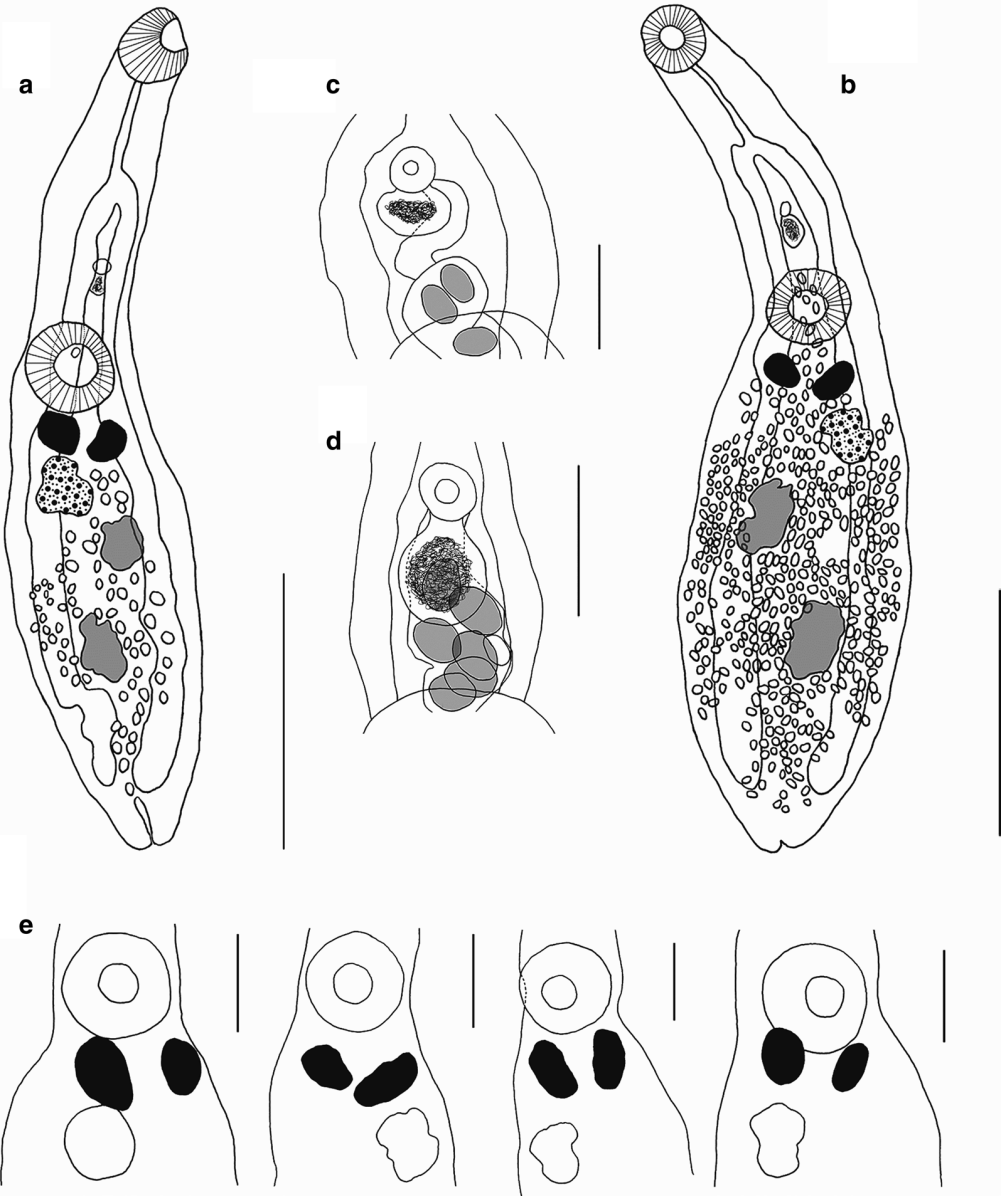
*Phyllodistomum macrocotyle* ex *Scardinius erythrophthalmus*. **a**, **b** Whole-mount ventral view. **c**, **d** Detail of the male genitalia. **e** Shape and position of vitelline glands. Scale-bars: **a**, **b** 500 μm; **c**, **d** 100 μm; **e** 200 μm

[Based on 11 gravid specimens; Fig. 4]. Body elongate, with smooth lateral margins, 1764–2277 (1943 ± 150) long, 180–720 (442 ± 141) wide. Forebody cylindrical, 621–7773 (707 ± 75) long, 33–41% (36%) of total body length. Hindbody lanceolate, 1026–1404 (1227 ± 126) long, 56–67% (63%) of total body length. Oral sucker subterminal, round or sometimes oval, 176–258 (201 ± 28) long, 138–234 (178 ± 25) wide. Ventral sucker round or oval, 150–306 (189 ± 44) long, 150–306 (191 ± 43) wide. Oral sucker length to width ratio 1:0.78–1.25 (1:1.1). Ventral sucker length to width ratio 1:0.61–1.08 (1:0.95). Oral sucker to ventral sucker distance 462–654 (543 ± 60). Pharynx absent. Oesophagus long, straight, 90–240 (160 ± 44) long. Intestinal bifurcation 258–510 (363 ± 64) from anterior end. Caeca terminating close to the posterior extremity, 42–252 (144 ± 53).

Testes 2, slightly lobed, oblique. Anterior testis 84–162 (132 ± 26) long, 51–132 (106 ± 23) wide; posterior testis 120–204 (164 ± 33) long, 64–156 (116 ± 64) wide. Posterior testis to ovary distance 252–366 (303 ± 33). Seminal vesicle saccular, comparatively short, 37–99 (71 ± 16) long, 29–66 (46 ± 11) wide. Pars prostatica not observed. Genital pore median, midway between caecal bifurcation and ventral sucker margin, 66–180 (127 ± 32) from anterior margin of ventral sucker.

Ovary irregular, faintly lobed, sinistral in 8 specimens and dextral in 3; 117–150 (134 ± 11) long, 75–162 (107 ± 75) wide. Vitelline masses 2, compact, entire, round, oval or irregular, between ovary and ventral sucker, right vitelline mass 84–126 (106 ± 19) long, 48–90 (63 ± 13) wide, left vitelline gland 86–192 (118 ± 37) long, 55–96 (73 ± 13) wide. Uterus extensively coiled, occupying entire hindbody, inter- and extracaecal. Eggs oval, 29–33 × 11–22 (31 × 19). Excretory vesicle I-shaped, extending to level of caeca end.

### Remarks

*Phyllodistomum macrocotyle* morphologically closely resembles *P. elongatum.* This species can be differentiated from *P. elongatum* by the lobed nature of the vitelline masses, together with a shorter oesophagus, intercaecal uterus and a more posteriorly situated genital pore (near to ventral sucker margin).

### Tegumental topography of *Phyllodistomum macrocotyle*

**Fig. 5:**
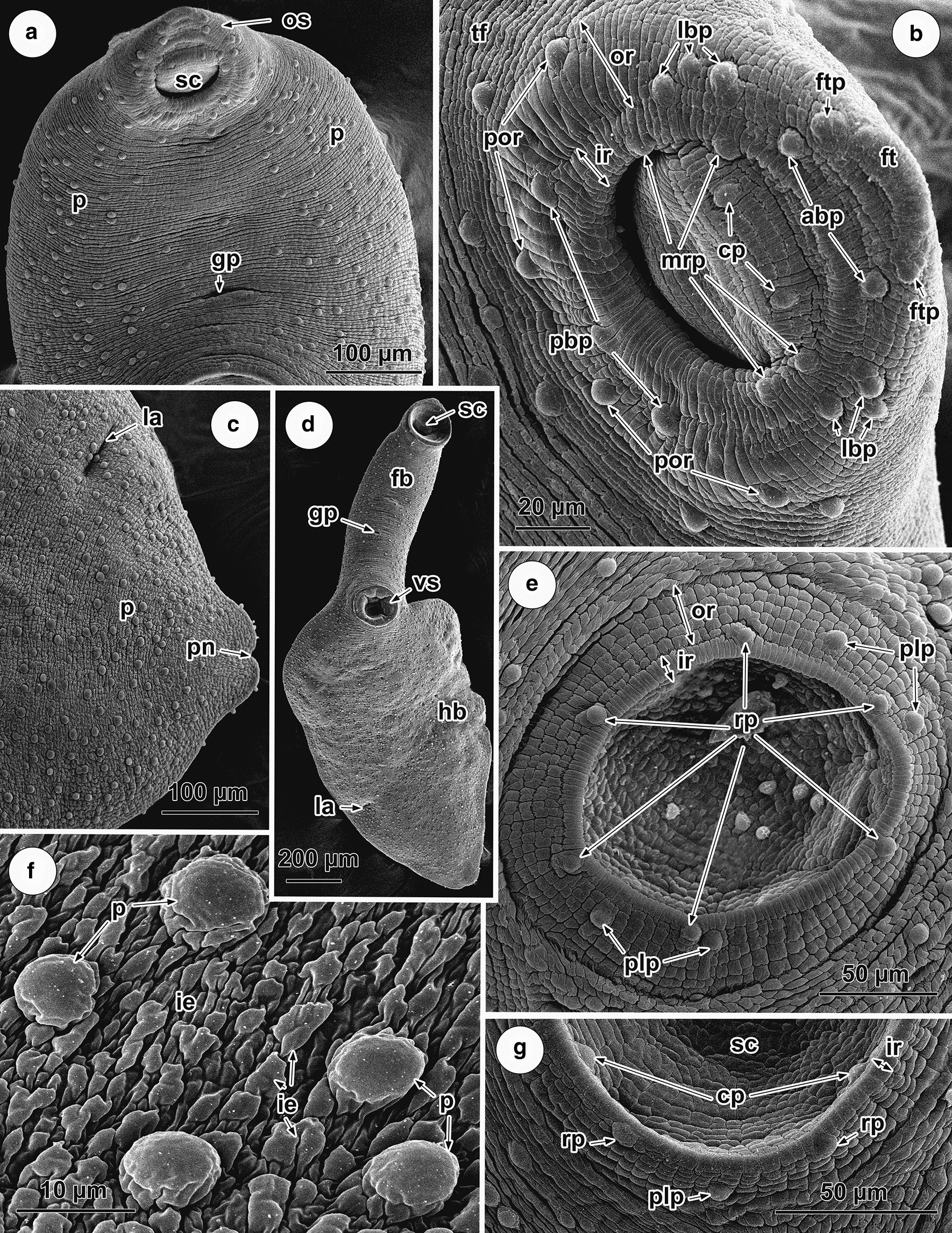
SEM micrographs of the ventral surface topography of adult *Phyllodistomum macrocotyle*. **a** Forebody of an unrelaxed worm, note the genital pore and randomly distributed papillae along the ventro-lateral surface. **b** Oral sucker showing two surrounding rings of tegument bearing surface papillae in a definite pattern. **c** Posterior extremity of the worm, note the pore of Laurer’s canal and the posterior notch. **d** Entire body of a relaxed worm. **e** Ventral sucker showing two surrounding rings of tegument bearing papillae in a definite pattern. **f** Button-like surface papillae, note the surrounding small, irregular surface elevations. **g** Region of the ventral sucker margin showing papillae within sucker cavity. *Abbreviations*: abp, apical papillae on border of sucker rim; cp, papillae within sucker cavity; fb, forebody; ft, frontal tubercle; ftp, papillae adjacent to frontal tubercle; gp, genital pore; hb, hindbody; ie, irregular elvations; ir, inner ring (rim) of oral sucker; la, pore of Laurer’s canal; lbp, lateral papillae on border of sucker rim; mrp, papillae on inner margin of sucker rim; or, outer ring of the oral and ventral suckers; os, oral sucker; p, papillae; pbp, posterior papillae on border of sucker rim; plp, postero-lateral papillae on outer ring of ventral sucker; pn, posterior notch; por, papillae on outer ring; rp, papillae on outer border of rim ventral sucker; sc, sucker cavity; tf, transverse tegumental folds; vs, ventral sucker

SEM analysis shows that there is a distinct irregular transverse folding of the body surface; this is especially clear in unrelaxed worms (Fig. 5a, b). Numerous small, irregular and shallow elevations were apparent on the tegumental surface at a greater magnification (Fig. 5f). A large number of papillae are scattered on the ventral surface of both the forebody and hindbody (Fig. 5a, c, d). These button-like papillae are rounded in shape, unciliate, possess a smooth surface and are 7.5–8.0 µm in diameter. They are distributed randomly on the surface of the forebody and are accumulated mainly on the ventro-lateral surface, with only solitary papillae being observed ventro-medially (Fig. 5a). Large number of these papillae is randomly distributed throughout the surface of the ventral hindbody (Fig. 5c, d).

The genital pore is situated ventro-medially in the forebody closer to the ventral sucker than to the oral sucker (Fig. 5a). The surrounding tegument is devoid of papillary aggregations (Fig. 5a). The slit-like pore of Laurerʼs canal is located on the ventral surface, about 2/3 of the distance between the ventral sucker and the posterior extremity (Fig. 5c). The excretory pore opens on a notch situated at the posterior extremity of the body (Fig. 5c).

The oral sucker is directed antero-ventrally (Fig. 5a, d). There are two superficial rings of tegument surrounding its aperture, both of which are characterised by radially directed surface corrugations of differing lengths and widths (Fig. 5b). The inner ring (rim) of the oral sucker is about 14 µm in width and possesses tighter corrugations when compared with the outer wider ring (~ 25 µm) which has larger corrugations. Twenty-one distinct, button-like regular papillae occur on the rings of the oral sucker and within its cavity. Within the sucker cavity there are two papillae (cp) localised antero-dorsally (Fig. 5b). Four papillae (mrp) (two on each side of the rim) are distributed laterally on the inner margin of the rim (Fig. 5b); 11 papillae occur close to the border between the inner and outer rings, two (abp) of which are localised apically juxtaposed to the papillae adjacent to the frontal tubercle (see below), six (lbp) are situated laterally (three on each side) and three (pbp) occur posteriorly (Fig. 5b); and four (por) are present posteriorly on the surface of the outer ring (Fig. 5b). Slightly antero-dorsal to the outer ring of the oral sucker is a distinct frontal tubercle with a single lateral papilla on each side (Fig. 5b).

The ventral sucker is also surrounded by two rings characterised by morphologically distinct surface layers (Fig. 5e). The inner ring (rim) (~10–12 µm in width) borders the sucker cavity and bears radially oriented corrugations, whereas the surface of the outer ring (~ 28–35 µm in width) has a densely packed, cuboidal structure (Fig. 5e). This sucker bears 14 papillae aggregated in a definite pattern (Fig. 5e): six uniformly distributed papillae (rp) are radially arranged and situated on the outer border of the rim (Fig. 5e); four of the same morphology and size (plp) occur on the outer ring arranged in two symmetrical pairs postero-laterally (Fig. 5e); and four similar papillae are randomly distributed within the sucker cavity (Fig. 5g).

## Discussion

Molecular comparison of specimens of *Phyllodistomum*, parasitizing *P. fluviatilis*, with respect to other congeners revealed significant genetic differences and reinforces the establishment of the new species. The parasite fauna of perch in Europe is relatively well studied, but *Phyllodistomum* spp. are rarely reported from this fish. As far as we know, the European perch hosts *P. pseudofolium*, *P. folium* and *P. angulatum* (see [23–26]). Unfortunately, identification of species of *Phyllodistomum* based solely on morphological characteristics has been unreliable and many of the records of these species from other fish hosts are questionable. The validity of five European *Phyllodistomum* spp., i.e. *P. folium*, *P. umblae*, *P. angulatum*, *P. pseudofolium* and *P. macrocotyle*, has been confirmed based on molecular markers. According to comparative molecular data *P. simile* Nybelin, 1926, a parasite of bull-head, showed no differences from *P. folium*, and *P. megalorchis* is to be regarded as a synonym of *P. angulatum*. Most species of *Phyllodistomum* have their own characteristic host associations and the levels of their host specificity are distinct. The type-species of the genus *Phyllodistomum*, *P. folium*, is euryxenous and its host specificity appeared the lowest among the known *Phyllodistomum* species [6]. This low host specificity was confirmed on the basis of molecular markers for *P. folium* specimens obtained from several fish hosts. This species was detected in *Esox lucius* (Esociformes, Esociformes), *Rutilus rutilus*, *Aspius aspius*, *Abramis ballerus*, *A. brama* (Cypriniformes, Cyprinidae), *Gymnocephalus cernuus* (Perciformes, Percidae), *Cottus gobio* (Scorpaeniformes, Cottidae), *Gasterosteus aculeatus* (Gasterosteiformes, Gasterosteidae) [5, 6]. Our results expand the host range of this species to another cyprinid fish, *S. erythrophthalmus.*

The present material permits comparisons between *P. elongatum*-like specimens recovered from different fish species. In our previous molecularly-based study, trematodes from ureters of *A. bramae* identified as *P. elongatum*, showed no differences from *P. folium* [5]. Here, based on sequence data we show that specimens of *Phyllodistomum* spp. recovered from ureters and urinary bladder of *S. erythrophthalmus* represents two different species, *P. macrocotyle* and *P. folium*, respectively. *Phyllodistomum* from ureters of *L. idus* proved to be *P. macrocotyle*, while *Phyllodistomum* from ureters of *R. rutilus* were identified as *P. folium*. Thus, it is evidently that *P. elogatum*-like trematodes localized in ureters of cyprinid fishes, can represent multiple species.

*Phyllodistomum* spp. are known for their considerable variation in features, such as egg size, sucker ratio, and body shape, features considered as diagnostic in other genera. Many of these features are severely affected by fixation techniques, flattening, and the condition of the worm at fixation (see [27]). According to Kudinova [1] the body shape of the *Phyllodistomum* species depends on the fish host species and on the morphology of its excretory system (form and size of the urinary bladder and ureters). Based on morphometric analysis of abundant material from different fish hosts Kudinova [1] concluded that the validity of only three *Phyllodistomum* species can be confirmed, namely, *P. conostomum* (syn. *P. umblae*), *P. folium* and *P. angulatum*, while *P. pseudofolium*, *P. simile*, *P. macrocotyle* (usually localised in ureters, rarely in the urinary bladder) and *P. dogieli* are just morphotypes of *P. folium*. The study of Namuleno & Scholz [28] revealed a great intraspecific variability of *P. folium* obtained from the urinary bladder of its type-host *Esox lucius*; only the diameter of suckers and their ratio and egg size appeared to be relatively stable characteristics. The shape of the body of *P. folium* differed markedly and quite different morphological types were found, from relatively slender, elongate or lanceolate flukes to broadly pyriform, with distinctly separated fore- and hindbody. Bakke [27] reported polymorphism in *P. umblae* from coregonid fish and recognized three different basic types of body shape.

The main consequence of the intraspecific phenotypic variation is an unclear view of the *Phyllodistomum* diversity in the European populations of fish. Earlier keys to European species of *Phyllodistomum* are uncritical and the authors approach to the diversity and validity of species is very different. Dawes [29] has stated that in the ultimate analysis all the European species of *Phyllodistomum* may become synonyms of *P. folium*. On the contrary, Pigulevsky [10] distinguished the nominal species, many of which are poorly known and inadequately described, by features that tend to be susceptible to treatment during or prior to fixation or to ontogenetic variation. He divided the genus *Phyllodistomum* into a number of subgenera; however, none of the subgenera proposed have received general acceptance [2]. Evaluating more than 1500 specimens of *P. folium*-like specimens, he concluded that the nominal species *P. folium* (Olfers, 1816) comprises four independent species, *P. folium*, *P. dogieli* Pigulevsky, 1953, *P. bychowskii* Pigulevsky, 1953 and *P. pseudofolium*; flukes from perciform fishes he attributed to *P. pseudofolium*. The validity of the new taxa erected by Pigulevsky [10] has been questioned by various authors. According to Bykchovskaya & Kulakova [23] descriptions of *P. dogieli*, *P. bychowskii*, *P. baueri* Pigulevski, 1953, *P. massino* Pigulevski, 1953 and *P. zachwatkin* Pigulevsky, 1953 (these species are listed in the database of Fauna Europaea [30]) are incomplete and their validity is doubtful.

Species of *Phyllodistomum* are relatively unusual among trematodes in having different types of cercariae which utilize highly diverse bivalve families, indicating that host extensions have featured in their histories [31–33]. However, there is no obvious morphological basis for distinguishing adults of *Phyllodistomum* spp. which develop from different cercariae, but it was presumed that these cercarial groups reflect phylogenetic distinctions [33]. Phylogenetic analyses of the family showed the genus *Phyllodistomum* to be paraphyletic and distinct clades correspond variously to the identity of the first intermediate host and the type of cercariae [34]. Recently *Phyllodistomum* sp. from unionids and common carp were recorded in Japan, the first record of rhopalocercariae (with comparatively short, club-shaped tail and absence of stylet and pharynx) in this country. Interestingly, it was noted that the morphology of the adult specimens resembles that of *P. elongatum* recorded in Europe [35]. It is worth noting that experimental studies on the life-cycle of European cercaria of rhopalocercous type *Cercaria duplicata* von Baer, 1827 from freshwater mussels yielded conflicting results; the study of Orechia et al. [36] has demonstrated that it is the larval form of *P. elongatum*, while Ivantsiv & Kurandina [37] showed that it is *P. angulatum*. However, molecular data revealed no match between *C. duplicata* and any species of *Phyllodistomum* [5].

The present phylogenetic analyses demonstrated that the new species, *P. kupermani* n. sp., is a member of a clade containing freshwater species with cystocercous cercariae developing in bivalves of the family Sphaeriidae. Morphologically, the new species most closely resembles *P. pseudofolium*, a highly host-specific parasite of *Gymnocephalus cernuus*. Our phylogenetic analyses showed that that *P. kupermani* n. sp. is genetically distantly related to *P. pseudofolium*, which produces macrocercous (but not cystocercous) cercariae developing in sphaeriid bivalve *Pisidium amnicum*. It should be noted that *P. pseudofolium*, *P. angulatum* and *P. macrocotyle* formed a highly supported clade despite the fact that these species appear to be associated with distinct patterns of first intermediate host identity and cercarial morphology [6].

Comparative analysis of the available data on the detailed tegumental topography of *Phyllodistomum* spp. clearly indicates that the SEM is a powerful tool in the discrimination and identification of closely related species. The surface topography of *P. macrocotyle* revealed in the present study differs from that described for congeneric species in the number and arrangement of papillae on both of the suckers and the surface of the body. Only one papillary type, button-like unciliated papillae, was observed. Large numbers of such papillae are randomly scattered along ventro-lateral regions of the forebody and on the entire ventral hindbody with no tendency to be concentrated in longitudinal rows or form other regular patterns. This differs from most other species of *Phyllodistomum* studied using SEM, i.e. *P. umblae*, *P. folium*, *P. inecoli* Razo-Mendivil, Pérez-Ponce de León, Rubio-Godoy, 2013, *P. spinopapillatum* Pérez-Ponce de León, Pinacho-Pinacho, Mendoza-Garfias & García-Varela, 2015, *P. pseudofolium* and *P. angulatum*, where one or two paired longitudinal rows of regular papillae are arranged on the surface of the forebody between the suckers [6, 8, 38–41]. However, as in *P. macrocotyle,* randomly distributed ventro-lateral papillae have been recorded for a recently described species, *P. wallacei* Pérez-Ponce de León, Martinez-Aquino & Mendoza-Garfias, 2015, a parasite of cyprinodontiform freshwater fish in central Mexico [4], although other papillary patterns in these two species differ. It is worth noting that irregularly arranged papillae have been described on the hindbody of all *Phyllodistomum* species, but their number varies significantly among the different species, from a few in *P. umblae* and *P. folium* (see [38, 40]), to a large number in *P. macrocotyle* and *P. wallacei* (see [4]; present study).

A great diversity in the patterns of papillae associated with the oral sucker has been described in species of *Phyllodistomum*. In the present SEM investigation of *P. macrocotyle*, 21 regular papillae were revealed in two rings around the oral sucker, which is a different papillary topography to that found around the oral sucker in all other species of *Phyllodistomum* examined to date. For example, a total of 16 papillae occur on the inner, upper, middle and lower parts of the oral sucker in *P. spinopapillatum* (see [8]); a consistent, bilaterally symmetrical arrangement of 18 papillae was noted on the rim and within the oral sucker of *P. umblae* (see [38]); in *P. wallacei* seven pairs of papillae have been described on the oral sucker (see [4]), in *P. angulatum* this figure was 20 papillae and in *P. pseudofolium* 16 [6].

There is a diversity in the location and number of papillae found on the surface of the ventral sucker of different *Phyllodistomum* species. In the present study, 14 papillae are aggregated in a definite pattern in *P. macrocotyle*, whereas in *P. folium*, *P. umblae*, *P. angulatum* there are 10 regular papillae [6, 38–40], in *P. cribbi* Pérez-Ponce de León, Martinez-Aquino & Mendoza-Garfias, 2015 and *P. wallacei* six papillae [4] and in *P. spinopapillatum* 18 regular papillae are associated with this sucker [8]. Nevertheless, despite differences in the overall number of papillae, in most species of this genus, there are six papillae on the rim of the ventral sucker and four papillae within the sucker [6, 8, 38–40].

## Conclusions

The present study illustrates the challenge of identifying closely related parasites that have poor morphological distinguishing features and had historically been described based solely on morphological characters, and emphasising the need to use molecular tools for accurate species identification and to provide insights into the evolution and radiation of such parasites. The molecular markers showed that the European perch, *P. fluviatilis*, hosts a new species of *Phyllodistomum*, *P. kupermani* n. sp., morphologically closely resembling *P. pseudofolium*. However, phylogenetic analysis shows that the new species is most closely related to the type-species of the genus, *P. folium*. The identity of other *Phyllodistomum* spp., reported in this fish, should be confirmed on the basis of molecular markers. Comparative molecular studies have also revealed that *P. elongatum*-like trematodes, recovered from ureters *S. erythrophthalmus* and *L. idus* represents *P. macrocotyle*, while *Phyllodistomum* specimens from ureters of *R. rutilus* were *P. folium*.

## Data Availability

*Phyllodistomum kupermani* n. sp. specimens were deposited in Parasite Collection of the Institute for Biology of Inland Waters RAS, Russia, holotype No. 1/13 (1), paratypes: No. 1/13 (2–6). Eleven voucher specimens of *P. macrocotyle* ex *S. erythrophthalmus* on 8 slides [No. 1/12(1–8)] were deposited in the Parasite Collection of the Institute for Biology of Inland Waters RAS, Russia. Nucleotide sequences obtained in the present study have been deposited into the GenBank database under accession numbers listed in Table 1.

## Abbreviations

GTR + G: general time reversible model with gamma distributed rate variation among sites
ITS2: internal transcribed spacer 2
ML: maximum likelihood
LM: light microscopy
SEM: scanning electron microscopy
SPR: subtree pruning and regrafting

## References

[1] Kudinova MA, Shulman SS (1994). On the revision of system of the trematode genus *Phyllodistomum* Braun, 1899 (Gorgoderidae). Ecological Parasitology.

[2] Campbell RA, Bray RA, Gibson DI, Jones A (2008). Family Gorgoderidae looss, 1899. Keys to the Trematoda.

[3] Nakao M (2015). *Phyllodistomum kanae* sp nov (Trematoda: Gorgoderidae), a bladder fluke from the Ezo salamander *Hynobius retardatus*. Parasitol Int..

[4] Pérez-Ponce de León G, Martinez-Aquino A, Mendoza-Garfias B (2015). Two new species of *Phyllodistomum* Braun, 1899 (Digenea: Gorgoderidae), from freshwater fishes (Cypriniformes: Goodeinae) in central Mexico: An integrative taxonomy approach using morphology, ultrastructure and molecular phylogenetics. Zootaxa..

[5] Petkevičiūtė R, Stunžėnas V, Stanevičiūtė G, Zhokhov AE (2015). European *Phyllodistomum* (Digenea, Gorgoderidae) and phylogenetic affinities of *Cercaria duplicata* based on rDNA and karyotypes. Zool Scripta..

[6] Stunžėnas V, Petkevičiūtė R, Poddubnaya L, Stanevičiūtė G, Zhokhov A (2017). Host specificity, molecular phylogeny and morphological differences of *Phyllodistomum pseudofolium* Nybelin, 1926 and *Phyllodistomum angulatum* Linstow, 1907 (Trematoda: Gorgoderidae) with notes on Eurasian ruffe as final host for *Phyllodistomum* spp. Parasit Vectors..

[7] Ho HW, Bray RA, Cutmore SC, Ward S, Cribb TH (2014). Two new species of *Phyllodistomum* Braun, 1899 (Trematoda: Gorgoderidae looss, 1899) from great barrier reef fishes. Zootaxa..

[8] Pérez-Ponce de León G, Pinacho-Pinacho CD, Mendoza-Garfias B, García-Varela M (2015). *Phyllodistomum spinopapillatum* sp nov (Digenea: Gorgoderidae), from the Oaxaca killifish *Profundulus balsanus* (Osteichthyes: Profundulidae) in Mexico, with new host and locality records of *P. inecoli*: morphology, ultrastructure and molecular evidence. Acta Parasitol..

[9] Petkevičiūtė R, Kudlai O, Stunžėnas V, Stanevičiūtė G (2015). Molecular and karyological identification and morphological description of cystocercous cercariae of *Phyllodistomum umblae* and *Phyllodistomum folium* (Digenea, Gorgoderidae) developing in European sphaeriid bivalves. Parasitol Int..

[10] Pigulevsky SW, Skryabin KI (1953). Family Gorgoderidae Looss, 1901. Trematodes of animals and man.

[11] Peribáñez MA, Ordovás L, Benito J, Benejam L, Gracia MJ, Rodellar C (2011). Prevalence and sequence comparison of *Phyllodistomum folium* from zebra mussel and from freshwater fish in the Ebro River. Parasitol Int..

[12] Stunžėnas V, Petkevičiūtė R, Stanevičiūtė G (2011). Phylogeny of *Sphaerium solidum* (Bivalvia) based on karyotype and sequences of 16S and ITS1 rDNA. Cent Eur J Biol..

[13] Petkevičiūtė R, Stunžėnas V, Stanevičiūtė G (2014). Differentiation of European freshwater bucephalids (Digenea: Bucephalidae) based on karyotypes and DNA sequences. Syst Parasitol..

[14] Bowles J, Blair D, McManus DP (1995). A molecular phylogeny of the human schistosomes. Mol Phylogenet Evol..

[15] Cribb TH, Anderson GR, Adlard RD, Bray RA (1998). A DNA-based demonstration of a three-host lifecycle for the Bivesiculidae (Platyhelminthes: Digenea). Int J Parasitol..

[16] Scholz T, De Chambrier A, Kuchta R, Littlewood DTJ, Waeschenbach A (2013). *Macrobothriotaenia ficta* (Cestoda: Proteocephalidea), a parasite of sunbeam snake (*Xenopeltis unicolor*): example of convergent evolution. Zootaxa..

[17] Tkach V, Grabda-Kazubska B, Pawlowski J, Swiderski Z (1999). Molecular and morphological evidences for close phylogenetic affinities of the genera *Macrodera*, *Leptophallus*, *Metaleptophallus*, and *Paralepoderma* (Digenea, Plagiorchioidea). Acta Parasitol..

[18] Olson PD, Cribb TH, Tkach VV, Bray RA, Littlewood DTJ (2003). Phylogeny and classification of the Digenea (Platyhelminthes: Trematoda). Int J Parasitol..

[19] Thompson JD, Higgins DG, Gibson TJ (1994). CLUSTAL W: improving the sensitivity of progressive multiple sequence alignment through sequence weighting, position-specific gap penalties and weight matrix choice. Nucleic Acids Res..

[20] Posada D (2008). JModelTest: phylogenetic modelling averaging. Mol Biol Evol..

[21] Tamura K, Peterson D, Peterson N, Stecher G, Nei M, Kumar S (2011). MEGA5: Molecular Evolutionary Genetics Analysis using maximum likelihood, evolutionary distance, and maximum parsimony methods. Mol Biol Evol..

[22] ICZN. *International Commission on Zoological Nomenclature*: Amendment of articles 8, 9, 10, 21 and 78 of the International Code of Zoological Nomenclature to expand and refine methods of publication. Bull Zool Nomencl. 2012;69:161–169.

[23] Bykhovskaya-Pavlovskaya IE, Kulakova AP, Bauer ON (1987). Trematoda. Key to parasites of freshwater fish of USSR.

[24] Rauckis E. Fish parasites in Lithuanian waters. Vilnius: Mokslas; 1988. **(In Russian)**.

[25] Niewiadomska K. Fish parasites in Poland. Digenea.Warszawa: Polskie Towarzystwo Parazytologiczne; 2003. **(In Polish)**.

[26] Kirjušina M, Vismanis K. Checklist of the parasites of fishes of Latvia. FAO Fisheries Technical Paper No. 369/3. Rome: FAO; 2007.

[27] Bakke TA (1988). Morphology of adult *Phyllodistomum umblae* (Fabricius) (Platyhelminthes, Gorgoderidae): the effect of preparation, killing and fixation procedures. Zool Scripta..

[28] Namuleno G, Scholz T (1994). Biometrical and morphological variability of *Phyllodistomum folium* (Olfers, 1816) (Trematoda: Gorgoderidae), a parasite of pike (*Esox lucius*). Helminthologia..

[29] Dawes B (1946). The Trematoda.

[30] Gibson DI. Fauna Europaea: Gorgoderidae, *Phyllodistomum*. In: Fauna Europaea version 2020.06. https://fauna-eu.org/cdm_dataportal/taxon/57108945-7276-463d-9e1b-f2b6d218a87d. Accessed 15 Jun 2020.

[31] Goodchild CG (1943). The life history of *Phyllodistomum solidum* Rankin, 1937, with observations on the morphology, development and taxonomy of the Gorgoderinae (Trematoda). Biol Bull..

[32] Fischthal JH (1951). Rhopalocercariae in the trematode subfamily Gorgoderinae. Am Midl Nat..

[33] Cribb TH (1987). A new species of *Phyllodistomum* (Digenea: Gorgoderidae) from Australian and New Zealand freshwater fishes with notes on the taxonomy of *Phyllodistomum* Braun, 1899. J Nat Hist..

[34] Cutmore SC, Miller TL, Curran SS, Bennett MB, Cribb TH (2013). Phylogenetic relationships of the Gorgoderidae (Platyhelminthes: Trematoda), including the proposal of a new subfamily (Degeneriinae n subfam). Parasitol Res..

[35] Urabe M, Ishibashi R, Uehara K (2015). The life cycle and molecular phylogeny of a gorgoderid trematode recorded from the mussel *Nodularia douglasiae* in the Yodo River. Japan. Parasitol Int..

[36] Orecchia P, Paggi L, Castagnolo L, Della Seta G, Minervini R (1975). Ricerche sperimentali sul ciclo biologico di *Phyllodistomum elongatum* Nybelin, 1926 (Digenea: Gorgoderidae Looss, 1901). Parassitologia..

[37] Ivantsiv VV, Kurandina DP (1985). Life cycle of *Phyllodistomum angulatum* (Trematoda, Phyllodistomidae). Vest Zool..

[38] Bakke TA (1984). A redescription of adult *Phyllodistomum umblae* (Fabricius) (Digenea, Gorgoderidae) from *Salvelinus alpinus* (L) in Norway. Zool Scripta..

[39] Bakke TA (1985). *Phyllodistomum conostomum* (Olsson, 1876) (Digenea, Gorgoderidae): a junior subjective synonym for *P umblae* (Fabricius, 1780). Zool Scripta..

[40] Bakke TA, Ždárská Z (1985). Tegumental microtopography and arrangement of papillae in adult *Phyllodistomum folium* (Olfers, 1816) (Digenea: Gorgoderidae) from pikes (*Esox lucius* L). Folia Parasitol..

[41] Razo-Mendivil U, Pérez-PoncedeLeón G, Rubio-Godoy M (2013). Integrative taxonomy identifies a new species of *Phyllodistomum* (Digenea: Gorgoderidae) from the twospot livebearer, *Heterandria bimaculata* (Teleostei: Poeciliidae), in Central Veracruz, Mexico. Parasitol Res..

[42] Petkevičiūtė R, Stunžėnas V, Stanevičiūtė G (2004). Cytogenetic and sequence comparison of adult *Phyllodistomum* (Digenea: Gorgoderidae) from the three-spined stickleback with larvae from two bivalves. Parasitology..

[43] Stunžėnas V, Cryan JR, Molloy DP (2004). Comparison of rDNA sequences from colchicine treated and untreated tissues. Parasitol Int..

[44] Rosas-Valdez R, Choudhury A, Perez-Ponce de Leon G (2011). Molecular prospecting for cryptic species in *Phyllodistomum lacustri* (Platyhelminthes, Gorgoderidae). Zool Scripta..

[45] Cutmore SC, Cribb TH (2018). Two species of *Phyllodistomum* Braun, 1899 (Trematoda: Gorgoderidae) from Moreton Bay. Australia. Syst Parasitol..

[46] Tkach V, Pawlowski J, Mariaux J. Phylogenetic analysis of the suborder Plagiorchiata (Platyhelminthes, Digenea) based on partial lsrDNA sequences Int J Parasitol. 2000;30:83–93. 10.1016/s0020-7519(99)00163-0 10675749

[47] Bolek MG, Snyder SD, Janovy JJ (2009). Alternative life cycle strategies and colonization of young anurans by *Gorgoderina attenuata* in Nebraska. J Parasitol..

[48] Petkevičiūtė R, Stunžėnas V, Stanevičiūtė G (2012). Clarification of the systematic position of *Cercariaeum crassum* Wesenberg-Lund, 1934 (Digenea), based on karyological analysis and DNA sequences. J Helminthol..

[49] Galaktionov KV, Blasco-Costa I, Olson PD (2012). Life cycles, molecular phylogeny and historical biogeography of the ‘pygmaeus’ microphallids (Digenea: Microphallidae): widespread parasites of marine and coastal birds in the Holarctic. Parasitology..

